# Whole‐transcriptome defines novel glucose metabolic subtypes in colorectal cancer

**DOI:** 10.1111/jcmm.18065

**Published:** 2023-12-20

**Authors:** Shaohua Li, Wei Fang, Jianfeng Zheng, Zhiqiang Peng, Biyue Yu, Chunhui Chen, Yuting Zhang, Wenli Jiang, Shuhui Yuan, Lingqiang Zhang, Xueli Zhang

**Affiliations:** ^1^ The Third School of Clinical Medicine Southern Medical University Guangzhou China; ^2^ Department of General Surgery Southern Medical University Affiliated Fengxian Central Hospital Shanghai China; ^3^ State Key Laboratory of Proteomics National Center for Protein Sciences (Beijing), Beijing Institute of Lifeomics Beijing China; ^4^ School of Life Sciences Hebei University Baoding China

**Keywords:** CRC, glucose, immune therapies, molecular subtype, SFRP2, THBS2

## Abstract

Colorectal cancer (CRC) is the most prevalent malignancy of the digestive system. Glucose metabolism plays a crucial role in CRC development. However, the heterogeneity of glucose metabolic patterns in CRC is not well characterized. Here, we classified CRC into specific glucose metabolic subtypes and identified the key regulators. 2228 carbohydrate metabolism‐related genes were screened out from the GeneCards database, 202 of them were identified as prognosis genes in the TCGA database. Based on the expression patterns of the 202 genes, three metabolic subtypes were obtained by the non‐negative matrix factorization clustering method. The C1 subtype had the worst survival outcome and was characterized with higher immune cell infiltration and more activation in extracellular matrix pathways than the other two subtypes. The C2 subtype was the most prevalent in CRC and was characterized by low immune cell infiltration. The C3 subtype had the smallest number of individuals and had a better prognosis, with higher levels of NRF2 and TP53 pathway expression. Secreted frizzled‐related protein 2 (SFRP2) and thrombospondin‐2 (THBS2) were confirmed as biomarkers for the C1 subtype. Their expression levels were elevated in high glucose condition, while their knockdown inhibited migration and invasion of HCT 116 cells. The analysis of therapeutic potential found that the C1 subtype was more sensitive to immune and PI3K‐Akt pathway inhibitors than the other subtypes. To sum up, this study revealed a novel glucose‐related CRC subtype, characterized by SFRP2 and THBS2, with poor prognosis but possible therapeutic benefits from immune and targeted therapies.

## 1 INTRODUCTION

Colorectal cancer (CRC) represents a significant global public health challenge, ranking as the third most common cancer and the second leading cause of cancer‐related mortality worldwide.^1^ While genetic risk factors account for a small percentage (12%–35%) of CRC cases, most are sporadic and influenced by modifiable environmental factors.^2^ Among the environmental factors, diet and nutrition have been recognized as key determinants of CRC risk and progression.^3^ In particular, glucose metabolism, which serves as the central hub of macronutrient metabolism, has been implicated in CRC pathogenesis and prognosis.^4^, ^5^

Glucose metabolism is essential for providing energy and biosynthetic precursors for cell growth and survival. However, cancer cells often exhibit altered glucose metabolism, characterized by increased glucose uptake and glycolysis, even in the presence of oxygen. This phenomenon, known as the Warburg effect, confers several advantages to cancer cells, such as resistance to hypoxia, evasion of apoptosis, and modulation of the tumour microenvironment.^6^ Previous studies have demonstrated that hyperlipidemia being associated with colorectal adenoma, and Type 2 diabetes increasing the risk of CRC by 26%–50%.^7^, ^8^, ^9^, ^10^, ^11^ Several genes and proteins involved in glucose metabolism, such as glucose transporters^12^, ^13^, ^14^ and glycolytic enzymes^12^, ^15^, ^16^ have been found to be dysregulated in CRC and associated with tumour aggressiveness and poor prognosis. CRC is closely related to the disorder of glucose metabolism, and the molecular mechanisms are very complex. The altered metabolism could influence both cancer cells and tumour environment. On one hand, the balance of self‐renewal and anti‐apoptotic abilities of CRC‐stem cells is influenced by glucose metabolism through several signalling pathways, including WNT, Hippo, and PI3K/Akt pathways.^17^, ^18^, ^19^ On the other hand, metabolic wastes as well as molecules regulating glycolysis can affect the function of immune cells in direct or indirect ways.^20^

However, CRC is a highly heterogeneous disease, cancer cells undergo adaptive changes as the disease progresses. Despite the growing evidence of the role of glucose metabolism in CRC, the molecular mechanisms and clinical implications of glucose metabolic heterogeneity in CRC remain poorly understood. Therefore, it is of great interest to identify and characterize CRC subtypes with distinct glucose metabolic signatures, and to explore their potential therapeutic targets and strategies.

Here, we employed the non‐negative matrix factorization (NMF) consensus clustering to identify CRC subtypes with specific glucose metabolism signatures.^21^ We screened out 2228 carbohydrate metabolism‐related genes from the GeneCards database, and further survival analysis identified 202 genes as prognosis genes in CRC according to the clinical information from the TCGA database. Based on the expression patterns of 202 glucose genes related to prognosis, we obtained three metaclusters in CRC by using NMF clustering. Notably, one of these subclusters, C1, exhibited poor survival outcomes and increased immune infiltration compared with the other two subtypes. The Kyoto Encyclopedia of Genes and Genomes (KEGG) and Gene Ontology (GO) analysis indicated that upregulated genes in C1 were specifically enriched in angiogenesis and cell adhesion‐related factors. Further analysis implicated secreted frizzled‐related protein 2 (SFRP2) and thrombospondin‐2 (THBS2) as key regulators for the C1 metacluster. And it was confirmed that both SFRP2 and THBS2 knockdown suppressed migration and invasion of HCT 116 cells under high glucose conditions. Furthermore, we evaluated the therapeutic potential of each subtype using the Genomics of Drug Sensitivity in Cancer (GDSC) database, and found that C1 might benefit from immune and PI3K‐Akt axis target therapies. Our study provides novel insights into the glucose metabolic subtypes and their clinical relevance in CRC, and suggests new avenues for personalized and precision medicine.

## 2 MATERIALS AND METHODS

### 2.1 Data acquisition

We obtained the processed mRNA expression data of colon adenocarcinoma and rectum adenocarcinoma (COADREAD) from the Cancer Genome Atlas (TCGA) database (https://portal.gdc.cancer.gov/), which included 647 tumour samples and 51 normal samples. We also retrieved the expression profiles and survival information of patients with CRC from the Gene Expression Omnibus (GEO) database of the National Center for Biotechnology Information (NCBI) using the Series Matrix File of GSE39582 (*n* = 562) and GSE17536 (*n* = 177), both of which were annotated using GPL570. We collected a total of 2228 carbohydrate metabolism‐related genes from the database GeneCards (https://www.genecards.org/) by setting Relevance score threshold at >3 for subsequent analysis.

### 2.2 Classification of glucose metabolism subtypes in CRC

We used the glucose metabolism‐related genes obtained from GeneCards as the total candidate gene set. We performed Cox regression analysis using the R package ‘survival’ to evaluate the association between each candidate gene and overall survival (OS). Then, we applied an unsupervised NMF clustering method using the NMF package. The optimal number of clusters was determined by selecting the value of k at which the correlation coefficient began to decline. Finally, the subtype assignment was verified using the mRNA expression data of the glucose‐metabolism‐related genes described above.

### 2.3 Performance verification of glucose metabolism subtypes

We used the limma package to analyse differentially expressed genes (DEGs) between metaclusters in CRC. Then, the genes with significantly different expressions in all possible comparisons were selected by setting corrected *p* at <0.05 and considered them subclass‐specific. We further selected the top 30 genes with the largest log_2_FC values in each subclass to create a prediction model. Finally, we compared the classification results obtained using the nearest template prediction (NTP) algorithm with those obtained using the NMF algorithm.^21^, ^22^

### 2.4 Analysis of immune cell infiltration

We used the microenvironment cell populations‐counter (MCP) algorithm to assess eight immune and two non‐immune stromal cell populations. The immune cell populations evaluated included T cells, CD8^+^ T cells, natural killer cells, cytotoxic lymphocytes, B cell lines, monocyte lineage cells, myeloid dendritic cells, and neutrophils. Meanwhile, the stromal cell populations evaluated were endothelial cells and fibroblasts. In addition, we applied the ESTIMATE algorithm to calculate immune scores and stromal scores, which reflected the abundance of stromal and immune cell genetic features.

### 2.5 Gene Set Enrichment Analysis (GSEA) in CRC metaclusters

We obtained the log_2_(Fold Change) values for each gene between the CRC subtypes using the limma package, while we performed GSEA analysis on the GO and KEGG pathway using the R package clusterProfiler. Finally, we selected the specific upregulated pathways (the top 10 pathways with the highest NES values) from each subtype for presentation. We obtained the gene sets from the MSigDB database (http://www.gsea‐msigdb.org/gsea/downloads.jsp).

### 2.6 Gene set variation analysis (GSVA)

GSVA is a non‐parametric unsupervised method for assessing the enrichment of transcriptomic gene sets. It converts gene‐level changes into pathway‐level changes by scoring the gene sets of interest to determine the biological function of the samples. In this study, we downloaded gene sets from the Molecular Signatures database and used the GSVA algorithm to comprehensively score each gene set to assess the potential biological functional changes of different samples.

### 2.7 Antibodies and reagents

The following antibodies were used in the study: anti‐SFRP2 (Santa Cruz, sc‐365,524, 1:300), anti‐THBS2 (Abcam, ab84469, 1:500), anti‐HSP90 (ABclonal, A5027, 1:2000) and anti‐GAPDH (ZSGB‐BIO, TA‐08, 1:2000). Secondary antibodies (Jackson Immuno Research Laboratories, West Grove, PA, USA) were diluted 1:4000 using 5% skim milk in Tris‐buffered saline.

### 2.8 Cell culture

The HEK293T and HCT116 cell lines were obtained from American Type Culture Collection (ATCC) and cultured in Dulbecco's Modified Eagle Medium (DMEM) media (high glucose) supplemented with 10% FBS and 1% penicillin–streptomycin antibiotics. For the different glucose concentration treatments, a glucose‐free DMEM complete medium was used to replace the high glucose DMEM medium for 8 h prior to treatment with the indicated glucose concentration (6 or 25 mM). All cells were maintained in a humidified incubator with 5% CO_2_ at 37 °C.

### 2.9 Lentivirus infection

The annealed oligonucleotide fragment was cloned into pGreenPuro plasmids to establish the pGreenPuro shRNA lentiviral vector. Stable clones of HCT116 cells were selected by culturing them in a medium containing puromycin for 48 h. The clones stably downregulating SFRP2 and THBS2 were identified and were further verified by western blotting. The shRNA sequences used in this study were SFRP2: 5’‐CGAGGAAGCTCCAAAGGTATGTGAA‐3′; and THBS2: 5′‐ GCAAGGACAAGACACACAACT −3′. The control construct (shNC) was made by inserting a luciferase sequence 5′‐GTGCGTTGTTAGTACTAATCCTATTT‐3′ into the pGreenPuro Lentivector.

### 2.10 Real‐time PCR

For mRNA analysis, 1 mL of TRIzol was used to extract total RNA of 3 × 10^6^ HCT116 cells, 1 μg of each RNA sample was reverse transcribed in a 10 μL reaction system following the manufacturer's instructions of the ReverTra AceTM qPCR RT Master Mix (Toyobo, FSQ‐201). The reverse transcription reaction was performed with one cycle of 15 min at 37°C, 5 min at 55°C, 5 min at 98°C on the ProFlex 3x32‐Well PCR System (Applied Biosystems, USA). Quantitative PCR was conducted on the ABI QuantStudio5 (Applied Biosystems, USA). For each sample, 1 μL of undiluted reverse‐transcribed cDNA was used in a 20 μL reaction system using 2 × RealStar Power SYBR qPCR Mix (GenStar, A311). The Maser Mix no‐RT Control (Toyobo, Osaka, Japan) was utilized in control experiments to test for possible contamination of genomic DNA in the RNA template, owing to the absence of reverse transcriptase. The relative abundance of target RNAs was normalized to the housekeeping gene GAPDH. The sequences of primers for specific genes used in RT‐PCR were as follows: SFRP2‐F: 5′‐CGAATACCAGAACATGCGGC‐3′; SFRP2‐R: 5′‐AACTTCTTGGTGTCCGGGTG‐3′. SFRP4‐F: 5′‐CGAACTCAAGTCCCGCTCAT‐3′; SFRP4‐R: 5′‐ ACTGTTCTCCGCTGTTCCTG‐3′. SPP1‐F: 5′‐GCCTCCTAGGCATCACCTGT‐3′; SPP1‐R: 5′‐GCCCATTTGTTGTTTGGCTG‐3′. THBS2‐F: 5′‐GGTCCGGAACACTGAAACCA‐3′; SFRP2‐R: 5′‐TGGTGACCAGCTTGCGTG‐3′.

### 2.11 Western blot

HCT 116 cells treated with different glucose concentrations (as described in Cell culture section) were collected and 5 × 10^6^ HCT 116 cells were lysed in 400 μL lysis buffer that consisted of 50 mΜ Tris–HCl, pH 8.0, 150 mΜ NaCl, 1% NP‐40, and 0.5% sodium deoxycholate and were heated with SDS‐PAGE loading buffer. For immunoblot analysis, all primary antibodies were diluted in the Primary Antibody Dilution Buffer (Beyotime, P0023). Secondary antibodies were diluted 1:4000 in 5% skim milk in Tris‐buffered saline.

### 2.12 Cell proliferation assay

Cells were counted and seeded on 96‐well plates (1600 cells per well) and incubated at 37°C with 5% CO2. Cell growth was assessed using Cell Counting Kit‐8 (Bimake, B34302) following the manufacturer's instructions. Cell numbers were measured every 24 h by adding 10% CCK‐8 solution to the wells 1 h before absorbance at 450 nm was detected. Each cell line was set up in three duplicate wells, and the experiment was repeated three times. Each data point represents a mean ± standard deviation (SD).

### 2.13 Wound healing assay

Wound healing assay was performed to evaluate the cell migration ability of different groups. 5 × 10^5^ cells were seeded to a well of 6‐well plate (NEST) in 1 mL culture media and incubated for 24 h. A scratch was made on the confluent monolayer cells to mimic a 350 μm wound using a 200 μL pipette tip. The cells were washed three times with PBS to remove the detached cells and debris. Fresh serum free medium was added to each well. Images of the scratch area were captured at 0, 24, and 48 h using Nikon Eclipse Ti2. The wound rate was calculated as the percentage of the initial wound area at each time point. The data were presented as mean ± SD of three independent experiments.

### 2.14 Cell migration and invasion assay

Cell migration assay was conducted in a 24‐well transwell plate with 8 μm polyethylene terephthalate membrane filters (Corning), according to the manufacturer's instructions as described previously.^23^ A cell suspension in serum‐free culture medium (5 × 10^4^) was added to the upper chamber, and each insert was placed in the lower chamber containing 10% FBS (Gemini) culture medium. Cells were allowed to migrate for 18 h in a humidified chamber at 37°C with 5% CO_2_. After 18 h, filters were fixed with 4% formaldehyde for 10 min, and cells in the lower filter were stained with 0.1% crystal violet for 10 min before being washed with clear water and imaged with Nikon Eclipse 50i. Three replicate wells were set up for each group, and the experiment was repeated three times. Each data point represents a mean ± SD.

### 2.15 Immunotherapy and drug sensitivity analysis

The SubMap analysis was applied to assess the similarity of gene expression profiles between our subclass and previously published patients treated with immunotherapy to predict the immunotherapy efficacy for the subclass. The largest pharmacogenomic database (GDSC Cancer Drug Susceptibility Genomics Database, https://www.cancerrxgene.org/) was used to predict the drug susceptibility of each tumour sample with the R package ‘pRRophetic’. Furthermore, regression was performed to obtain half maximal inhibitory concentration (IC_50_) estimates for each drug treatment, and 10 cross‐validations were conducted with the GDSC training set to test regression and prediction accuracy. All parameters were set to default values, including averaging duplicate gene expression and applying a ‘combat’ to remove batch effects.

### 2.16 Statistical analysis

The data analysis was performed using R software (version 4.0) and GraphPad Prism (version 9.5). The Kaplan–Meier method was used to generate survival curves, and log‐rank test was used to compare the survival differences between groups.^24^ Cox proportional hazards model was used to assess the hazard ratio (HR) with 95% confidence interval.^25^ The Kruskal–Wallis test was used for three‐group comparison.^26^ Statistical graphs in Figure 5 are prepared in Graphpad Prism, values were presented as mean ± standard deviation. For two group comparison, *p* values were derived from one‐way Student *t*‐test. All tests were two sided, and *p <* 0.05 was considered statistically significant.

## 3 RESULTS

### 3.1 Identification of metaclusters in CRC

**FIGURE 1:**
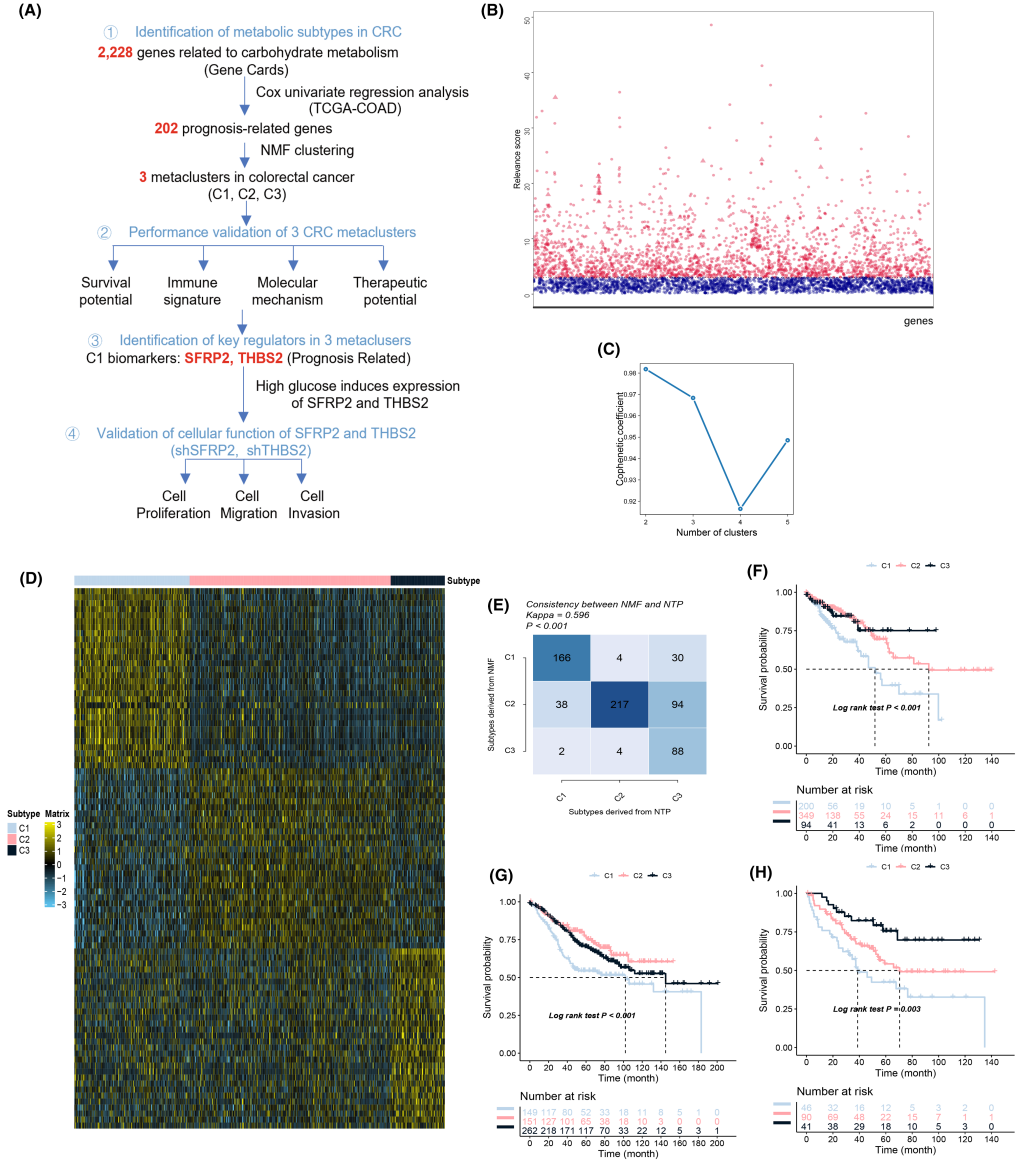
Identification of three glucose metabolism‐related subtypes of CRC. (A) The workflow employed in this study to identify metabolic subtypes and their key regulators in CRC. (B) Identification of 2288 carbohydrate metabolism‐related genes from the GeneCards database by setting the relevance score at 3. (C) Cophenetic correlation coefficients associated with different numbers of clusters k. (D) Heatmap showing the 90 biomarkers derived from the top 30 signature genes of each metacluster. (E) Comparison between NMF clustering and the NTP analysis based on 90 biomarkers. Cohen's kappa statistic is calculated to estimate the coefficient between NMF and NTP, the adjusted *p*‐value was obtained by the Benjamini–Hochberg procedure. (F–H) Survival analysis of the three metaclusters. Survival curves demonstrating the prognosis of C1, C2 and C3 subtypes from COADREAD patients in the TCGA database (F) and CRC dataset (GSE39582, GSE17536) from GEO database (G, H). The log‐rank test was used to test the survival distributions of C1, C2 and C3, the adjusted *p*‐value was obtained by the Benjamini–Hochberg procedure.

To classify CRC into distinct glucose metabolic subtypes, we developed a strategy to identify the key regulators in glucose metabolism that affect CRC prognosis, and then cluster CRC based on the expression pattern of the identified regulators (Figure 1A). First, 2228 carbohydrate metabolism‐related genes were retrieved from GeneCards with a Relevance score >3 cutoff (Figure 1B, Table S1). Further Cox analysis selected 202 genes with prognostic value according to the clinical information of 643 patients with CRC from the TCGA database (Table S2). Next, the NMF consensus clustering analysis was applied to analyse the expression profiles of the identified 202 genes in CRC cases and *k* = 3 was determined as the optimal number of clusters (Figure 1C). These clusters were named as C1, C2 and C3, and it was shown that each subtype had a unique enrichment of the top 30 genes with the largest log2FC values (Figure 1D). The robustness of the clustering was further confirmed by NTP clustering, which revealed a significant interconnectivity between the NTP and the NMF clustering methods (Figure 1E). Moreover, significant prognostic differences among the subtypes were noticed, especially for the C1 subtype, which had a worse survival outcome than C2 and C3 in the TCGA dataset (Figure 1F). Further survival analysis of two independent datasets from the GEO database confirmed the poorest survival potential for C1, while C2 and C3 exhibited relatively unstable survival outcomes. (Figure 1G,H). These data suggested that it is feasible and meaningful to classify and annotate CRC according to glucose metabolic genes, and that the C1 metacluster might represent a novel molecular subtype with poor prognosis in CRC.

### 3.2 The immune characteristics in CRC metaclusters

**FIGURE 2:**
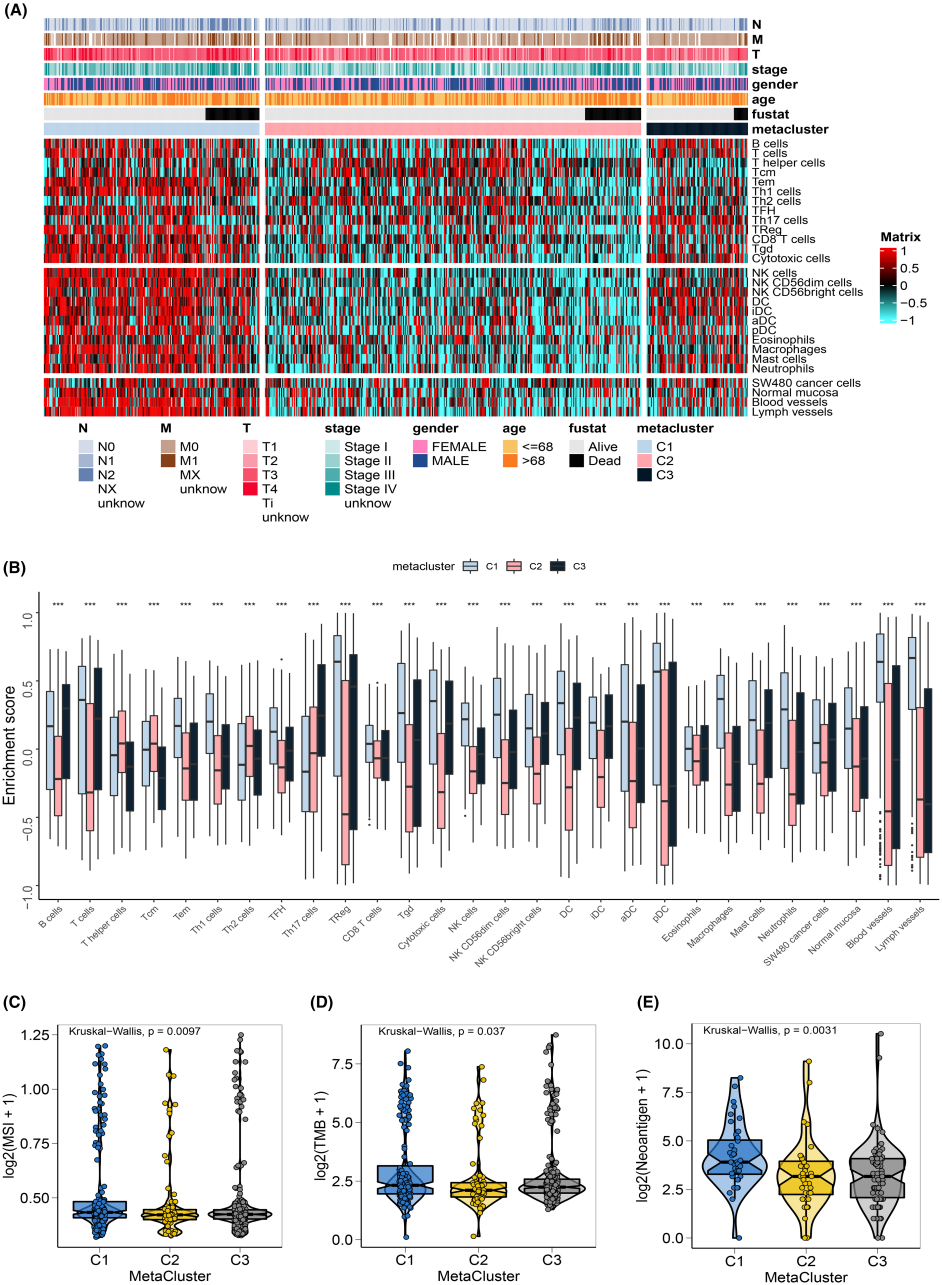
The immune signatures of CRC metaclusters. (A) Heatmap of immune characteristics of the three CRC metaclusters. (B) Boxplot indicating the enrichment scores of immune characteristics among the three metabolic subtypes. Violin diagram showing the immune score (C), stromal score (D) and tumour purity (E) of the three identified subclusters in CRC. The overall *p* values among the three data sets in (B–E), were calculated by the Kruskal–Wallis test, the *p* values between two data sets in (C–E) were obtained by the Wilcoxon test. *p < 0.05, **p < 0.01, ***p < 0.001.

Increased evidence suggests that metabolic reprogramming not only plays a crucial role in cancer cell growth, but also regulates anti‐tumour immune responses.^6^, ^27^ Since the altered microenvironment might inhibit the anti‐tumour function of immune cells, we explored the immune profiles in the identified subtypes of CRC. It turned out that the three metaclusters presented with distinct differences in their immune profiles (Figure 2A). The analysis of immune cells enrichment suggested that the C1 subtype scored higher than the C2 and C3 subtypes for most immune cell types, including CD8^+^ T, memory T cell, and follicular helper T cells. Notably, C2 scored significantly lower than the other two subtypes in most immune cell (Figure 2B). The tumour purity of the three subtypes was further analysed using the R package ‘ESTIMATE’, the results showed significantly higher immune and stromal scores in C1 than in C2 and C3 (Figure 2C,D), indicating a lower tumour purity in the C1 subtype than in C2 and C3 (Figure 2E).^28^ These findings suggested that the C1 subtype might maintain a more active immune profile compared to the other two metaclusters in CRC.

### 3.3 The metabolic subtypes of CRC exhibit unique molecular profile

**FIGURE 3:**
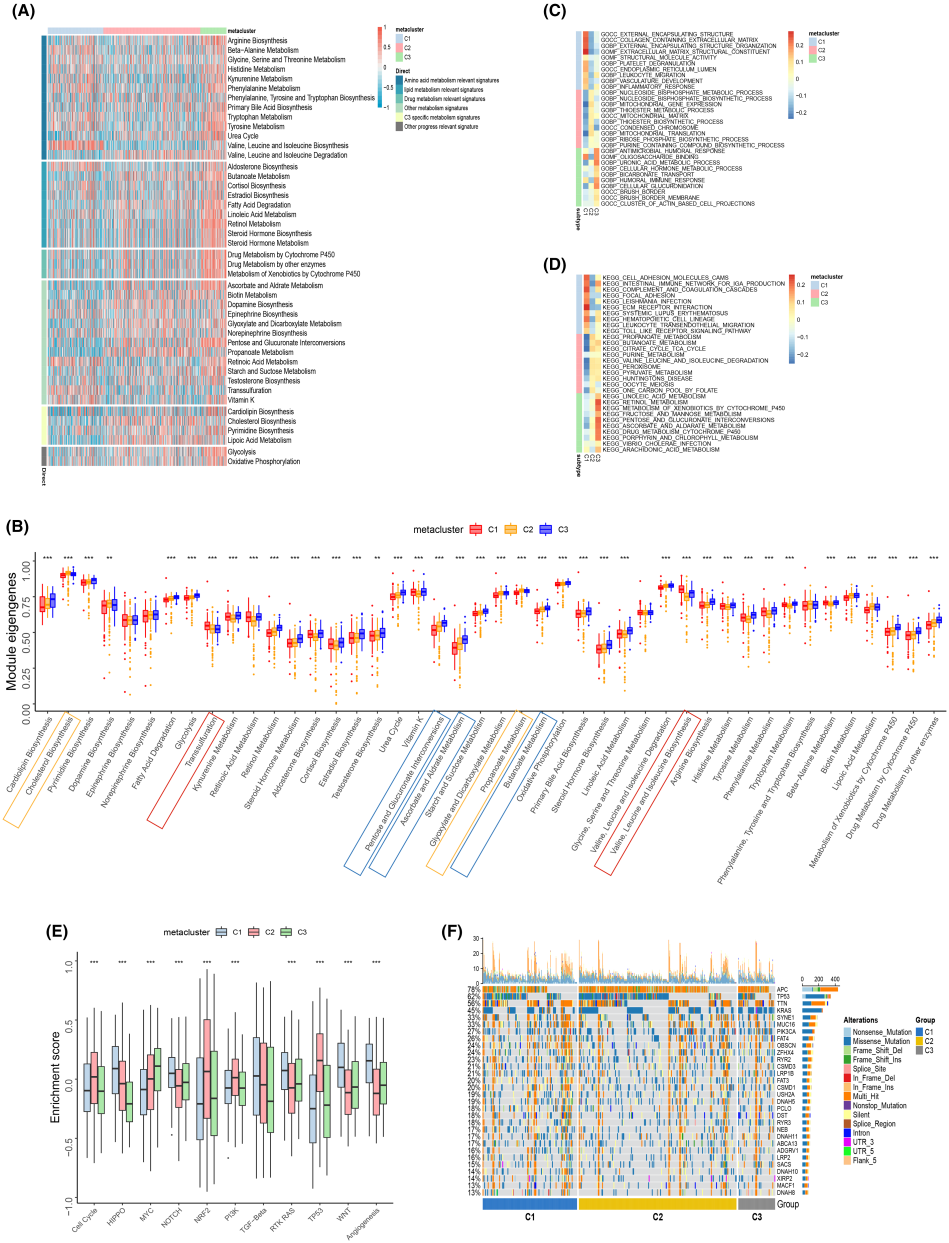
Molecular profile of CRC metabolic subtypes. Heat map (A) and box plot (B) showing the expressions of genes in metabolic pathways in the three metaclusters. (B) The black asterisk above the box plot indicates that there is a statistically significant difference in this pathway among the three subtypes. The pathways that specifically upregulated in C1, C2 and C3 are marked with red, yellow and blue boxes, respectively. The heat map presents the top 10 entries with GO (C) and KEGG (D) pathway analysis for each of the three. (E) Box plot comparing the enrichment of common pro‐oncogenic and anti‐oncogenic pathways in the three metaclusters in CRC. (F) The oncoplot shows the distribution of mutated genes in the identified CRC metaclusters. The *p*‐values in (B–E) were calculated by the Kruskal–Wallis test, *p < 0.05, **p < 0.01, ***p < 0.001.

Previous studies have found that metabolic reprogramming affects the progression of malignant diseases through various pathways.^29^ To explore the characteristic molecular mechanisms of the three metabolic subtypes of CRC, we quantified the signalling pathways in the three CRC metaclusters with ssGSEA algorithm. The results showed that the C1 subtype was more active in some amino acid metabolism pathways (transsulfuration, valine leucine isoleucine biosynthesis) than the other two subtypes, while lipoic acid metabolism, vitamin metabolism (Retinol Metabolism, ascorbate and aldarate metabolism) and pentose phosphate pathways were down‐regulated in C1 (Figure 3A,B). Furthermore, we performed GO and KEGG pathway analysis using the GSVA algorithm and found that the pathways enriched in the C1 subtype were mainly related to collagen containing extracellular matrix (ECM), external encapsulating structure organization and ECM structural constituent (Figure 3C). Similarly, the KEGG analysis in C1 was mainly enriched in cell adhesion molecules cams, intestinal immune network for IgA production, complement and coagulation cascades (Figure 3D). Further analysis of common tumour‐related pathways revealed that C1 scored higher than C2 and C3 in the HIPPO, NOTCH, RTK RAS, WNT and angiogenic pathways, C3 scored higher for NRF2 and TP53 pathways (Figure 3E). Specifically, the HIPPO, NOTCH, and WNT pathways had a positive regulatory effect on angiogenesis, indicating that the C1 subtype may have a specific ECM component and may be more advantageous in tumour angiogenesis. Furthermore, mutational profiling with the three metabolic subtypes in CRC demonstrated that the proportion of mutations in several genes, including APC, KRAS, and SYNE1, varied significantly between subtypes (Figure 3F).

Taken together, our data suggest that the C1 subtype of CRC may have specific metabolic properties and molecular pathways, primarily related to cell adhesion and angiogenesis, and this might be the reason behind the worse prognosis of C1 compared to C2 and C3 subtypes.

### 3.4 SFRP2 and THBS2 are biomarkers of the C1 metacluster

**FIGURE 4:**
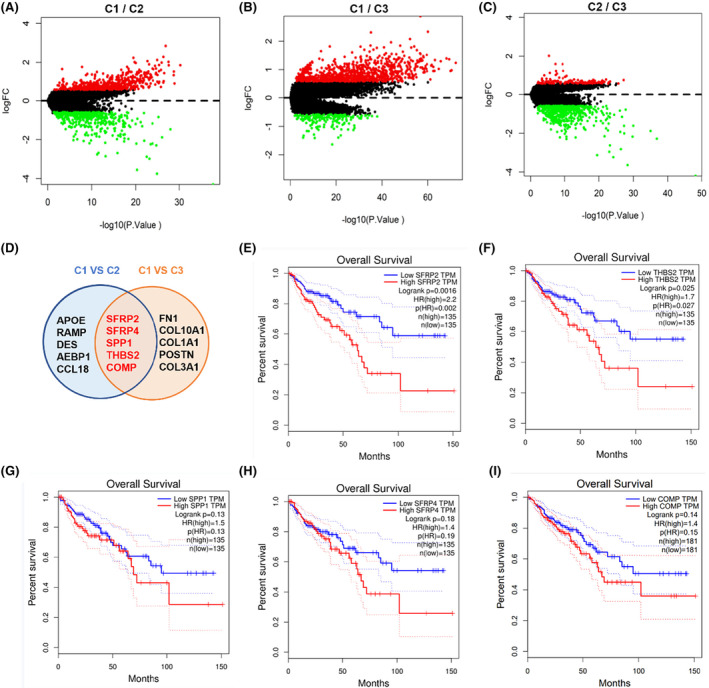
Identification of biomarkers in the three CRC metaclusters. (A) Volcano diagram showing differentially expressed genes (DEGs) between CRC subclusters C1 and C2. (B) Volcano diagram showing DEGs between CRC subclusters C1 and C3. (C) Volcano diagram showing DEGs between CRC subclusters C2 and C3. (D) The Venn diagram demonstrating the top 10 up‐regulated genes in the C1 compared to C2 and C3 metaclusters, respectively. (E–I) Survival analysis of indicated CRC patients based on GEPIA database. Kaplan–Meier Survival analysis comparing the survival potential of CRC patients with different SFRP2 (E), THBS2 (F), SPP1 (G), SFRP4 (H) and COMP (I) expression levels.

To further investigate the molecular markers for the three metaclusters, the DEGs (|logFC| > 0.585, *p* < 0.05) were identified between the subgroups (C1_vs_C2, C1_vs_C3, C2_vs_C3). An upregulation of 911 genes and a downregulation of 174 genes in C1 were noticed compared to C2 (Figure 4A). Similarly, 556 genes were upregulated and 441 genes were downregulated in the C1 when compared to the C3 cluster (Figure 4B). In the comparison of C2 and C3, 115 genes were highly expressed in C2 subtype and 579 genes were expressed highly in C3 (Figure 4C). As the C1 metacluster showed a worse prognosis, we focused on the signature genes of the C1subtype. The top 10 genes specifically upregulated in C1 in the comparisons of C1 versus C2 and C1 versus C3 were picked out. Interestingly, five genes merged in the two comparison and exhibited a particular higher expression in the C1 metacluster, including SFRP2, SFRP4, THBS2, SPP1 and COMP (Figure 4D). Further survival analysis of these five genes with Gene Expression Profiling Interactive Analysis (GEPIA) database revealed that high expression of SFRP2 and THBS2 were associated with a poor prognosis in patients with CRC (Figure 4E–I). These results suggested that SFRP2 and THBS2 might serve as biomarkers for the C1 subtype and could negatively regulate the prognosis of CRC.

### 3.5 SFRP2 and THBS2 promotes migration and invasion of CRC cells

**FIGURE 5:**
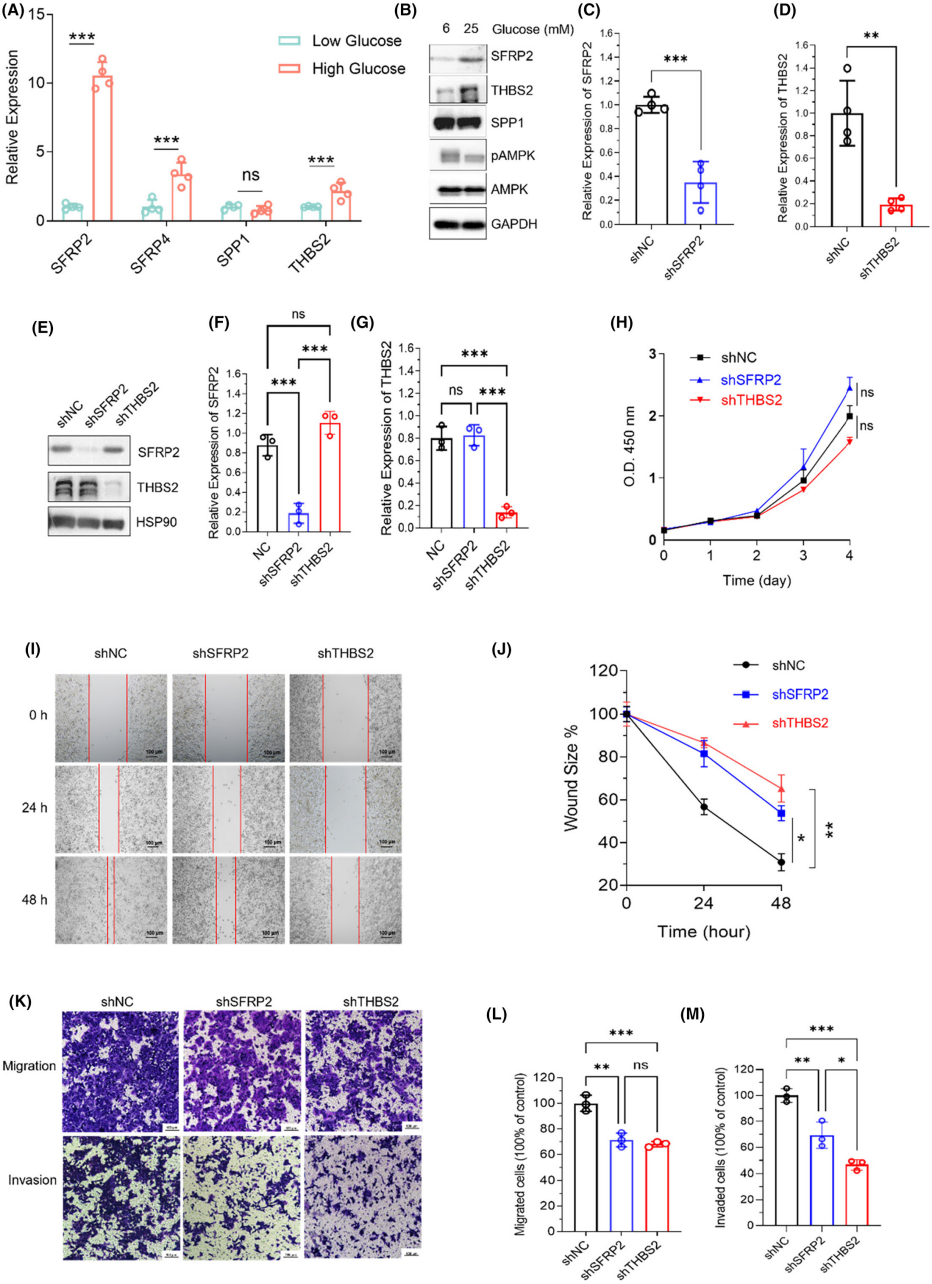
SFRP2 and THBS2 promote migration and invasion of HCT116 cells in high glucose condition. (A) The qRT‐PCR analysis of relative mRNA expression of SFRP2, SFRP4, SPP1 and THBS2 in HCT116 cells with low (6 mM) or high (25 mM) glucose concentration treatment. (B) Western Blot analysis of the expression of indicated proteins in HCT116 cells treated with 6 or 25 mM glucose. (C) qRT‐PCR analysis of the relative expression of SFRP2 in SFRP2 knockdown (shSFRP2) or negative control (NC) HCT116 cells. (D) qRT‐PCR analysis of the relative expression of THBS2 in THBS2 knockdown (shTHBS2) or negative control (NC) HCT116 cells. (E) Western blot analysis of the SFRP2, THBS2 and SPP1 protein expression in SFRP2 or THBS2 knockdown HCT 116 cells. (F) Grey scale quantification and statistical analysis of the blots of SFRP2 relative to HSP90 in (E). (G) Grey scale quantification and statistical analysis of the blots of THBS2 relative to HSP90 in (E). (H) The OD450 values of SFRP2 and THBS2 knockdown cells were analysed by CCK‐8 assay. (I) Representative images of wound healing assay in SFRP2 and THBS2 knockdown cells. (J) The quantification of migration rates of SFRP2 and THBS2 knockdown cells by measuring the wound size in three different areas. (K) Representative images of migrated cells and invaded cells in transwell migration and invasion assay. (L) Statistical analysis of migrated cells in transwell migration assay. (M) Statistical analysis of invaded cells in transwell invasion assay. Data represent the mean ± SD of three independent experiments. The Student *t*‐test was used to compare the two independent data sets in (A, C, D). Bonferroni adjusted for multiple comparisons in (F–H, J, L, M). *p < 0.05, **p < 0.01, ***p < 0.001.

In order to investigate the roles of SFRP2, SFRP4, THBS2, and SPP1 in abnormal glucose metabolism, we treated HCT 116 cells with different glucose concentrations and measured their expression levels using real‐time PCR. Interestingly, we found that treatment of 25 mM glucose induced the expression of SFRP2, SFRP4 and THBS2 compared to 6 mM glucose treatment, and no such effect was observed with SPP1 (Figure 5A). Further western Blot analysis confirmed the elevated expression of SFRP2 and THBS2 under high glucose conditions (Figure 5B). Given that SFRP2 and THBS2 have a negative effect on CRC prognosis (Figure 4E,F), we constructed SFRP2 and THBS2 knockdown HCT116 cell lines and validated the knockdown efficacy at the mRNA (Figure 5C,D; Figure S1) and protein levels (Figure 5E–G). The CCK8 analysis showed that the knockdown of neither THBS2 nor SFRP2 inhibited the proliferation of HCT116 cells (Figure 5H). However, wound healing assays indicated that the knockdown of either SFRP2 or THBS2 significantly inhibited migration in HCT116 cells (Figure 5I,J). The transwell experiments further confirmed that the ability of migration and invasion was impaired in SFRP2 or THBS2 knockdown HCT 116 cells (Figure 5K–M). The above data suggest that SFRP2 and THBS2 could be biomarkers in C1, and the expression of SFRP2 and THBS2 might be elevated in high glucose conditions and promote the migration and invasion of CRC cells.

### 3.6 The C1 metacluster benefits from immune and target therapy

**FIGURE 6:**
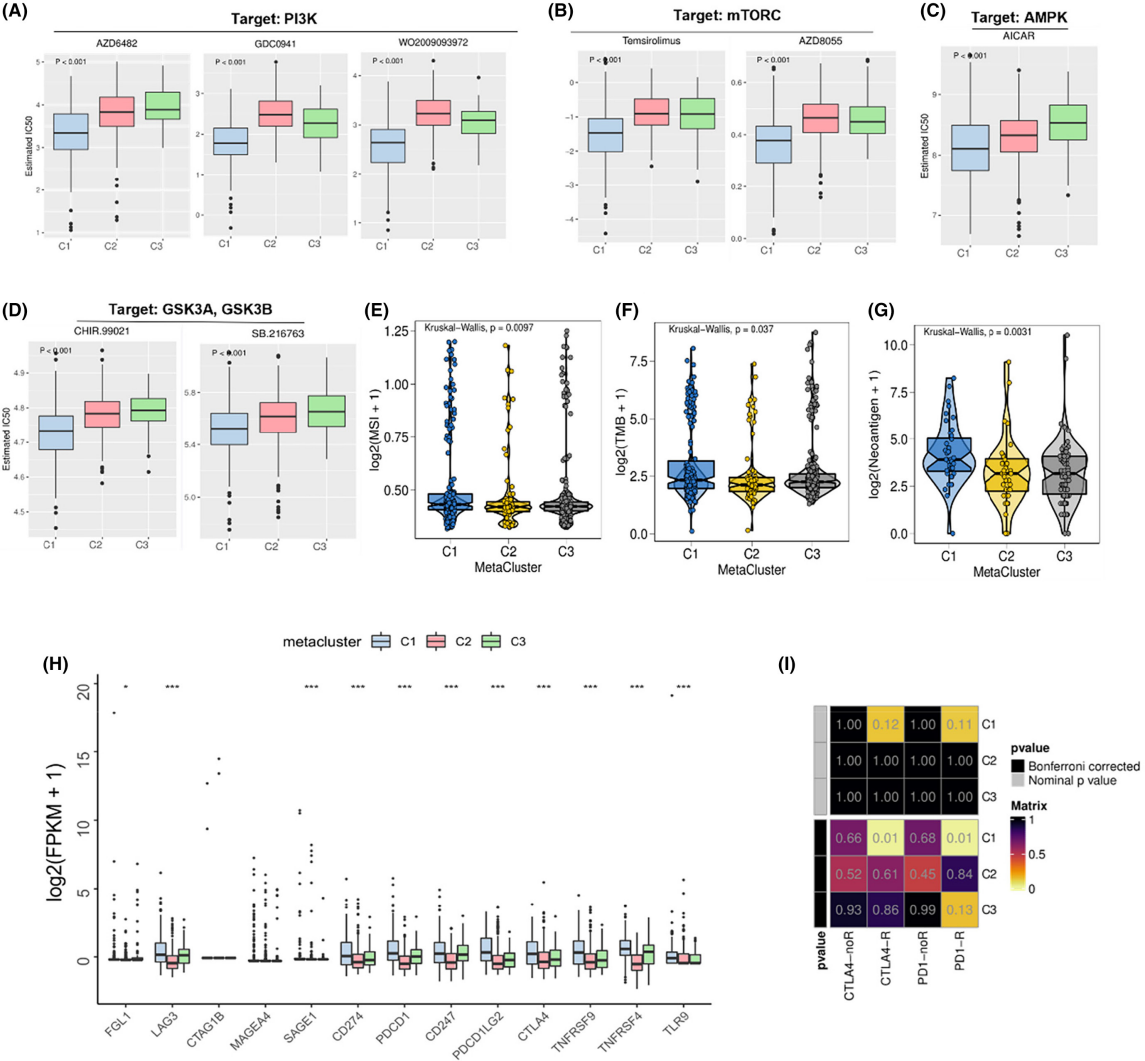
Therapeutic potential of targeted therapies and immunotherapy in three CRC metaclusters. (A–D) Estimated IC_50_ of indicated compounds in the three CRC subtypes. Box plots comparing the estimated IC_50_ of PI3K (A), mTORC (B), AMPK © and GSK3 (D) targeting therapy in the identified metaclusters in CRC. (E) Comparison of MSI levels in the three CRC subtypes. (F) The TMB scores in the three CRC subtypes. (G) The predicted neoantigens of the three CRC metaclusters. (H) The expression of the indicated immune checkpoints in the three CRC metaclusters. (I) Sensitivity analysis of three CRC metaclusters to antitumor immunotherapy with indicated targets. The *p* values in (A–I) were calculated by the Kruskal–Wallis test, *p < 0.05, **p < 0.01, ***p < 0.001.

To further explore the therapeutic potential of the three metabolic subtypes in CRC, we used the GDSC database and the R package ‘pRRophetic’ to explore the sensitivity of various drugs in each tumour sample. It turned out that compounds targeting key nodes in the PI3K/Akt/mTORC pathway, including PI3K, mTORC, AMPK and GSK3, exhibited relatively higher sensitivity in the C1 subtype compared to the C2 and C3 subtypes (Figure 6A–D). On the other hand, the microsatellite instability, neoantigen, and mutation burden levels of the C1 subtype were significantly higher than those of the C2 and C3 subtypes (Figure 6E–G). To further predict the efficacy of immunotherapy on different subtypes, we analysed the expression of 13 potential immune checkpoint genes in the three CRC metaclusters. The analysis of immune checkpoints revealed a higher expression of PDCD1LG2, CTLA4 and TNFRSF9 in the C1 subtype (Figure 6H). Furthermore, the analysis of immunotherapy potential predicted that the C1 subtype had a higher sensitivity to CTLA4 and PD1 immunotherapy than the other subtypes (Figure 6I). The above findings suggest that the C1 metacluster may benefit from targeted drugs for the PI3K/Akt pathway and exhibit a better response to specific immunotherapy.

## 4 DISCUSSION

Colorectal cancer is a complex disease that can be influenced by various environmental and genetic factors. As CRC is predominantly identified during its advanced stages, precise classification and targeted treatment are essential. The consensus molecular subtypes (CMSs) are currently the most robust classification system for CRC, including CMS1 (microsatellite instability immune, 14%), CMS2 (canonical, 37%), CMS3 (metabolic, 13%) and CMS4 (mesenchymal, 23%).^30^ Notably, the C1 subtype identified in our study shares similarities with CMS3 in overrepresenting KRAS mutation, which contributes to resistance to EGFR‐targeted therapy in metastatic CRC. Increasing studies indicate that tumours harbouring commonly assumed driver events in CRC still vary markedly in their biology, and identification of molecular subtypes in CRC may provide additional information for precise therapy.

Abnormal metabolism is a hallmark of cancer, with special energy requirements and excessive proliferation of tumour cells being mutually reinforcing. The malignant transformation of tumour cells promotes the evolution of metabolic pathways,^31^ while abnormal metabolic molecules, such as lactate and adipokines, act as oncogenic pathway regulators.^32^, ^33^, ^34^ CRC is a typical metabolism‐related cancer, and diabetes is the most common metabolic disease associated with an increased risk (~40%) for CRC. The molecular mechanism underlying this association may involve hyperinsulinemia and insulin resistance. Hyperinsulinemia or insulin resistance can promote CRC progression by activating insulin receptors or other related factors, such as insulin‐like growth factors (IGFs), sex hormones and adipokines. These abnormal conditions lead to the relative activation of epithelial cells to insulin, activating the insulin signalling pathway and promoting cancer cell proliferation and migration. Our study focuses on the relationship between glucose metabolism and CRC by classifying CRC into three glucose metabolism subtypes. Our findings provide a new insight for molecular mechanisms and therapeutic targets for glucose metabolism‐related CRC.

SFRP2 is a member of the Secreted Frizzled Related Protein family, whose well established function is to inhibit Wnt/β‐catenin signalling pathway by binding to Frizzled receptors, inhibiting cell proliferation, migration, and invasion.^35^ However, a growing number of studies have shown that SFRP2 is associated with metastasis, poor prognosis and treatment resistance in a variety of malignant diseases, including melanoma, prostate cancer and glioma.^35^, ^36^, ^37^, ^38^, ^39^ Mechanismly, this noncanonical function of SFRP2 might be explained by its interactions of regulators beyond Wnt pathways.^36^ A recent study suggested that SFRP2 promotes transition of glioblastoma to a mesenchymal subtype by suppressing SOX2,^39^ while another research found that SOX2 directly binds to the promoter of SFRP2, devoting to migration and invasion of CRC cells.^40^ These studies indicated that the roles of SFRP2 in cancers are complex, and border and deeper investigation about SFRP2 needs to be carried out. THBS2 belongs to the thrombospondin family and is a calcium‐binding glycoprotein that is mainly secreted by stromal cells into the ECM. It regulates numerous cell adhesion and migration events by binding to the ECM, cell receptors and growth factors.^41^ Previous studies demonstrated that knockdown of THBS2 inhibits progression and metastasis of CRC cell lines and affects the prognosis of CRC,^42^, ^43^ it was also found to present with elevated expressions in 17 types of cancer tissues compared with the corresponding adjacent normal tissues in a pan‐cancer study involving 38 types of malignancies.^44^ THBS2 was identified as a biomarker that distinguished tumours from normal tissues in multiple human cancers and a predictor for post‐surgical survival in patients with early‐stage lung adenocarcinoma.^45^, ^46^ Here, our study suggested that SFRP2 and THBS2 are not only related to CRC prognosis, but they are also functional regulators for migration and invasion of CRC cells.

Accumulating studies indicated that SFRP2 and THBS2 are correlated with metabolic dysregulation. The circulating insulin levels, as well as abnormal glucose tolerance and BMI, are positively correlated with serum SFRP2.^47^ THBS2 was also reported as a core gene in metabolic syndrome, a condition related to abnormal glucose and lipid metabolism.^48^, ^49^, ^50^ These findings indicate that SFRP2 and THBS2 may be vital regulators in abnormal glucose metabolism and CRC progression. Furthermore, as secreted proteins, SFRP2 and THBS2 can be detected by serology.^51^, ^52^ Unlike in situ materials, liquid samples are easy to obtain and can be used for early diagnosis and dynamic monitoring, providing a more comprehensive picture of the systemic status.^53^ Together, our study validated SFRP2 and THBS2 as key regulators that link abnormal glucose metabolism with CRC. However, further in‐depth and comprehensive studies on the clinical value of SFRP2 and THBS2 in CRC patients are warranted.

To sum up, our study demonstrated a novel clustering method for patients with CRC based on glucose metabolism. We classified CRC into three metaclusters using NMF clustering. Among these, the C1 subtype was associated with a worse survival potential, higher metastatic ability, abnormal cell adhesion, and angiogenesis. We identified SFRP2 and THBS2 as potential biomarkers for the C1 metacluster and showed that they promote CRC cell migration and invasion in response to high glucose stimulation. Further analysis suggests that C1 may respond better to CTLA4, PD1, PI3K, mTORC, and GSK3‐targeted therapies. Our findings highlight the importance of understanding the mechanism of glucose metabolism in CRC and provide potential strategies for precise therapy in CRC management.

## AUTHOR CONTRIBUTIONS

**Shaohua Li:** Conceptualization (equal); formal analysis (supporting); investigation (equal); software (supporting); validation (lead); writing – original draft (lead). **Wei Fang:** Investigation (equal); methodology (equal); validation (equal). **Jianfeng Zheng:** Data curation (supporting); visualization (equal). **Zhiqiang Peng:** Conceptualization (supporting); data curation (equal); methodology (equal). **Biyue Yu:** Resources (equal). **Chunhui Chen:** Formal analysis (equal); software (equal). **Yuting Zhang:** Resources (equal). **Wenli Jiang:** Resources (equal). **Shuhui Yuan:** Visualization (equal). **Lingqiang Zhang:** Conceptualization (equal); funding acquisition (equal); project administration (equal); supervision (equal); writing – review and editing (equal). **Xueli Zhang:** Conceptualization (equal); funding acquisition (equal); project administration (equal); supervision (equal); writing – review and editing (equal).

## CONFLICT OF INTEREST STATEMENT

The authors declare that they have no conflicts of interest for this work.

## Supporting information


Figure S1:

Table S1:

Table S2:

## Data Availability

The original contributions presented in the study are included in the article/Supplementary Material. Further inquiries can be directed to the corresponding author.
