# Supplementary material for: Whole‐transcriptome defines novel glucose metabolic subtypes in colorectal cancer
Source: J Cell Mol Med. 2023 Dec 20;28(5):e18065. doi: 10.1111/jcmm.18065 (PMC10902307; doi:10.1111/jcmm.18065)
Supplement: Supplementary file 1 — Figure S1: [file JCMM-28-e18065-s001.docx]

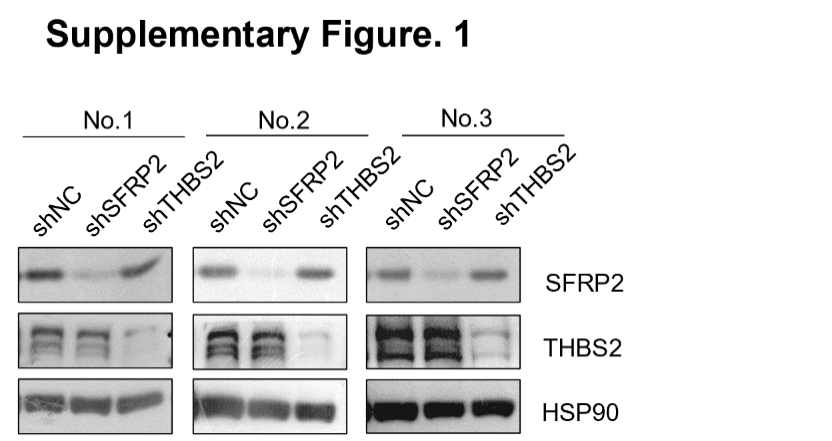
**Supplementary Figure 1**

**Supplementary Figure 1:** The SFRP2 and THBS2 knockdown efficacy

Western blot was performed to test the knockdown efficacy of shSFRP2 and shTHBS2 at protein level in three independent experiments.
